# Quercetin and Allopurinol Ameliorate Kidney Injury in STZ-Treated Rats with Regulation of Renal NLRP3 Inflammasome Activation and Lipid Accumulation

**DOI:** 10.1371/journal.pone.0038285

**Published:** 2012-06-11

**Authors:** Chuang Wang, Ying Pan, Qing-Yu Zhang, Fu-Meng Wang, Ling-Dong Kong

**Affiliations:** State Key Laboratory of Pharmaceutical Biotechnology, School of Life Sciences, Nanjing University, Nanjing, People’s Republic of China; Louisiana State University, United States of America

## Abstract

Hyperuricemia, hyperlipidemia and inflammation are associated with diabetic nephropathy. The NLRP3 inflammasome-mediated inflammation is recently recognized in the development of kidney injury. Urate and lipid are considered as danger signals in the NLRP3 inflammasome activation. Although dietary flavonoid quercetin and allopurinol alleviate hyperuricemia, dyslipidmia and inflammation, their nephroprotective effects are currently unknown. In this study, we used streptozotocin (STZ)-induced diabetic nephropathy model with hyperuricemia and dyslipidemia in rats, and found over-expression of renal inflammasome components NLRP3, apoptosis-associated speck-like protein and Caspase-1, resulting in elevation of IL-1β and IL-18, with subsequently deteriorated renal injury. These findings demonstrated the possible association between renal NLRP3 inflammasome activation and lipid accumulation to superimpose causes of nephrotoxicity in STZ-treated rats. The treatment of quercetin and allopurinol regulated renal urate transport-related proteins to reduce hyperuricemia, and lipid metabolism-related genes to alleviate kidney lipid accumulation in STZ-treated rats. Furthermore, quercetin and allopurinol were found to suppress renal NLRP3 inflammasome activation, at least partly, via their anti-hyperuricemic and anti-dyslipidemic effects, resulting in the amelioration of STZ-induced the superimposed nephrotoxicity in rats. These results may provide a basis for the prevention of diabetes-associated nephrotoxicity with urate-lowering agents such as quercetin and allopurinol.

## Introduction

Kidney injury is the most common pathological disorder predisposing end-stage renal disease worldwide [1]–[3]. Hyperuricemia, hyperlipidemia and inflammation are associated with the development and progression of diabetic kidney injury [2]–[6]. Uric acid contributes to the secretion of proinflammatory cytokines such as interleukin 1β (IL-1β) and IL-18 [7]. The elevated IL-1β and IL-18 levels are observed in diabetic patients with nephropathy [2], [8]–[10]. The NOD-like receptors (NLRs) are a family of intracellular sensors of danger-associated molecular patterns. NLR protein 3 (NLRP3, also known as NALP3/Cryopyrin) interacts with the bridging molecule apoptosis-associated speck-like protein (ASC) to activate caspase-1, which is essential for mature IL-1β and IL-18 production [7], [11], [12]. The NLRP3 inflammasome activation promotes kidney injury process with up-regulation of IL-1β and IL-18 [13], [14] and enhances IL-1β levels in the type 2 diabetes [15]. It is noted that high uric acid level is a causative factor of the NLRP3 inflammasome-mediated inflammation in lung injury [7]. Dyslipidemia is involved in the retarded urate clearance, causing the elevated serum uric acid level [16]. Free fatty acids synergistically enhance urate to activate the NLRP3 inflammasome [17], being responsible for obesity-induced inflammation and insulin resistance in fat depots of mice [18]. Therefore, serum level of uric acid is suggested as a new player in the development of diabetic nephropathy [5], [6]. Interestingly, allopurinol, an inhibitor of uric acid synthesis, reduces hemozoin-induced NLRP3 inflammasome activation [19] and controls IL-1β production in inflammasome-deficient mice [7]. The previous studies from our and other laboratories show that allopurinol reduces serum total cholesterol (TC) and triglycerides (TG) levels and ameliorates renal histopathology in fructose-fed rats [20], [21]. Allopurinol also exerts renoprotective action in the type 2 diabetic db/db mice [22] and patients with diabetic kidney injury [23]. These observations suggest that the NLRP3 inflammasome may be a therapeutic target for urate-lowering treatment in diabetes with kidney injury driven by hyperuricemia and dyslipidemia.

Dietary flavonoid quercetin is used as a hypouricemic agent [20]. Our previous study showed that quercetin attenuated fructose-induced hyperuricemia and renal dysfunction through the regulation of renal urate transport-related proteins such as short isoform of glucose transporter 9 (rGLUT9), renal-specific transporter (rRST), organic anion transporters 1 (rOAT1) and electrogenic urate transporter (rUAT) in rats [20]. Moreover, quercetin improves dyslipidemia and hyperinsulinemia in obese Zucker rats [24], fructose-fed rats [20] and atherogenic diet-fed mice with hepatic lipid accumulation [25], as well as reduces inflammation in visceral adipose tissue [25], primary human adipocytes [26] and the kidney of fructose-fed rats [20]. It also prevents diabetic kidney injury in rats [27]. However, the mechanisms by which quercetin and allopurinol protect against diabetic kidney injury are incompletely understood.

Streptozotocin (STZ) is known to develop diabetic kidney injury with hyperuricemia, hyperlipidemia and inflammation [6], [28]. The purpose of the present study was then to determine if the NLRP3 inflammasome was activated with the exacerbated production of IL-1β and IL-18 in the kidney of STZ-induced diabetic rats associated with high urate level and lipid metabolism abnormality, and conversely, if quercetin and allopurinol inhibited renal NLRP3 inflammasome activation involved in their actions on hyperuricemia and lipid metabolism disorder in this model.

## Materials and Methods

### Animals

Male Sprague-Dawley rats (5-week old and 200–220 g weight), were purchased from Laboratory Animal Center (Zhejiang Province, P. R. China). They were individually housed and maintained at a temperature of ∼22°C with a relative humidity of ∼55%, acclimatized for at least a week before further operation. Water and food were provided *ad lib* and light was set to a 12 h light/dark cycle (lights on at 8 a.m.). All protocols including diabetes induction and sacrifice operation had been approved by the Institutional Animal Care and Use Committee at the Nanjing University and the China Council on Animal Care at Nanjing University.

### Experimental Design

Rats were fasted overnight and then given a single intraperitoneal injection of freshly 55 mg/kg STZ (St Louis, MO, USA) in 0.1 M citrate buffer (pH 4.4) in a dark environment, while normal control animals were received only citrate buffer. The rats, whose fasting blood glucose levels in tail vein were above 250 mg/dL 72 h after STZ injection using glucose assay kit purchased from Jiancheng Biotech (Nanjing, P. R. China), were considered as diabetes. These successful model rats of diabetes were further divided into 5 groups (n = 8): diabetic control treated with water, diabetic rats treated with quercetin (25, 50 and 100 mg/kg), and allopurinol (10 mg/kg, a positive drug) (St Louis, MO, USA), respectively. The doses chosen were based on our and other previous preclinical experiments [29]–[32]. The treatment was started on 4th day after STZ injection by gavage once daily at 9∶00 a.m.–10∶00 a.m. for the subsequent 7 weeks.

### General Condition, Uric Acid and Kidney Function

Body weight and blood glucose levels of rats were recorded before and after STZ induction at each week, including drug treatment period. Blood samples were collected from rat tail vein, left to clot in pre-iced tubes and centrifuged at 3,000×*g*, 4°C for 5 min to get serum. Urine samples were collected with metabolic cages every week during drug treatment, and centrifuged at 3000×*g* for 5 min to remove the impurity. The samples were stored at −20°C for the assays. Serum and urine glucose levels were detected as above. Urinary creatinine (Cr), blood urea nitrogen (BUN) and albumin levels were determined by specific commercial kits purchased from Jiancheng Biotech (Nanjing, P. R. China). Uric acid (UA) levels in serum and urine were determined as previously reported [20]. Clearance rates of 24-h urine uric acid (Ucr), creatinine (Ccr) and BUN were calculated by the formula: Ucr/Ccr/BUN  =  urine UA/Cr/BUN concentration (µmol/L) × urine volume (L) in 24-h/serum UA/Cr/BUN (µmol/L)/1440.

### The Kidney-to-Body Weight Ratio and the Measurements of Serum and Tissue Samples

At the end of drug treatment, all animals were deprived of food but not water until the following morning. Rats were sacrificed by decapitation 1 h after the last administration between 9 a.m. and 10 a.m. Blood samples were collected as above. The kidney was quickly dissected and weighted (for calculation of the quot of kidney, the ratio of wet kidney weight/body weight). The left kidney cortex tissues were immediately frozen in liquid nitrogen.

The separated serum and kidney tissues were stored at −80°C for the measurements of TC, TG and non-esterified fatty acid (NEFA) levels by respective kits obtained from Jiancheng Biotech (Nanjing, P. R. China) and of IL-1β and IL-18 levels by ELISA kits (R & D).

### Histopathology

Kidney tissue from rats was immediately fixed for 1 day at room temperature in formalin and preserved in 70% ethanol. Renal biopsies were dehydrated with a graded series of alcohol and embedded in paraffin. Specimens were cut in 7-µm-thick section on a rotary microtome and mounted on APES-coated glass slide. Each section was deparaffinized in xylene, rehydrated in decreasing concentrations of alcohol in water and stained with hematoxylin-eosin (HE) reagent and periodic-acid schiff with diastase (PAS-D) reagent (Sigma Chemicals Co, St. Louis, MO), respectively. The slide was mounted with neutral balsam.

Other rat kidney tissues were snap-frozen immediately at −70°C. 6-µm-thick cryostat section was prepared on APES-coated glass slide. Each section was washed by distilled water and then stained with oil-red O reagent (Sigma Chemicals Co, St. Louis, MO) for 5–10 min. After washed with 60% isopropyl alcohol, the sections were re-stained by hematoxylin.

### Real-Time Quantitative PCR Analysis

Total RNA isolated from individual rat kidney with Trizol reagent (Invitrogen) following the manufacturer’s protocol, was to evaluate mRNA expressions of urate transport-related genes (rOAT1, OAT3, rUAT, rRST and rGLUT9), lipid metabolism-related genes [peroxisome proliferator-activated receptor-α (PPAR-α), carnitine palmitoyl transferase 1 (CPT1), organic cation/canitine transporter 2 (OCTN2), acetyl-CoA carboxylase 2 (ACC2)], NLRP3 inflammasome (rNLRP3, rASC, rCaspase-1) and glyceraldehyde 3-phosphate dehydrogenase (rGAPDH). The reverse transcribed cDNA using SuperScript First-Strand Synthesis kit (RT-PCR; Invitrogen). The primers used for Real-Time PCR were summarized in Table 1. All primer sequences were checked in GenBank to avoid inadvertent sequence homologies. They were designed and synthesized by Biogenes Biotechnology (Nanjing, R. P. China). Reactions were performed using SYBR-Green PCR master mix (Applied Biosystems) in a BioRad iCycler iQ Detection System. As an internal control, rGAPDH levels were quantified in parallel with target genes. Normalization and fold change for each of the genes were calculated using the 2-Delta Delta C (T) method [33].

**Table 1:** The primers used for Real-Time PCR.

Gene	Forward primer 5’→ 3’
	Reverse primer 5’→ 3’
rOAT1	TCATCTACTCTTGGTTCTTCATTG
	CGGAGCACCTCTATACTTAGC
rOAT3	GCTGGCTGGTTCTATCTG
	CTTGGCTGAGGTGATGTC
rUAT	CATCCACACAGTTCACAGCATCC
	GCAAGACCACGCCTGATATGTTG
rGLUT9	AGTCCTACTGCTTCCTCGTCTTTG
	CCTTGTTCCTCTTGGCGAATGC
rRST	GCTGCCTCTGCTGGTGTATGG
	TGGATGTCTTGGATGGTGTCAGG
rPPARα	GAGGCAGAGGTCCGATTCTTCC
	CGATCAGCATCCCGTCTTTGTTC
rCPT1	AAGTCAACGGCAGAGCAGAGG
	GGACACCACATAGAGGCAGAAGAG
rOCTN2	GAGACGAAGGACGGACGACAG
	TCACGATGGTGGACAGGAAGAC
rACC2	GAGGCACAGGTGAAGCAGGAG
	TCGGATAGTGGAACGCAGGTTG
rNALP3	CAGACCTCCAAGACCACGACTG
	CATCCGCAGCCAATGAACAGAG
rASC	TTATGGAAGAGTCTGGAGCTGTGG
	AATGAGTGCTTGCCTGTGTTGG
rCaspase-1	TGCCTGGTCTTGTGACTTGGAG
	ATGTCCTGGGAAGAGGTAGAAACG
rGAPDH	TGCACCACCAACTGCTTAG
	GATGCAGGGATGATGTTC

### Western Blot

About 100 mg of frozen rat kidney tissues were homogenized in 1 mL RIPA buffer, and then centrifuged at 10,000 × *g* for 20 min. Protein concentrations of the supernatants were measured by Bradford method. The total proteins were incubated in boiling water for 5 min. Equal amount of total protein was separated on 6–12% SDS-PAGE and electrophoretically transferred to polyvinylidene difluoride (PVDF) membrane (Millipore, Shanghai, P. R. China) pre-activated by methanol in the transferring buffer. Membranes were blocked with 5% skimmed milk for 2 h, and incubated overnight with specific primary antibodies at 4°C. Immunoreactive bands were detected using HRP-conjugated goat anti-rabbit or goat anti-rat IgG as the secondary antibody (1∶5,000) (Jingmei Biotech, Shanghai, P. R. China). Immunoreactive bands were visualized using phototope-horseradish peroxidase Western blot detection system (Cell Signaling Technologies, Beverly, MA) and quantified by densitometry using Molecular Analyst Software (Bio-Rad Laboratories, Hercules, CA). Primary antibodies included rabbit polyclonal antibodies against rOAT1 (1∶2,000), rOAT3 (1∶2,000), rUAT (1∶200) and rGLUT9 (1∶1000) (Sai-Chi Biotech. Beijing, P. R. China), rCPT1 (1∶3,000), rACC2 (1∶2,000), phosphorylation of rACC2 (p-rACC2, Ser219/Ser221, 1∶2,000) (Santa Cruz Biothchnology. CA), rPPAR-α (1∶2,000), rASC (1∶1,000), rCaspase-1 (1∶1,000) (Abcam Ltd. Hong Kong), rNLRP3 (1∶500) (Novus Biologicals, Inc. USA), rIL-1β (we detected both mature- and pro-IL-1β, 17 and 31 kD, respectively) (1∶1000) (Abcam Ltd. Hong Kong), rIL-18 (we detected both mature- and pro-IL-1β, 18 and 24 kD, respectively) (1∶500) (Santa Cruz Biothchnology. CA) and rat GAPDH (1∶5,000) (Kangcheng Biotech., Shanghai, P. R. China).

Renal cortical brush-border membrane vesicles were prepared from rat kidneys as previously reported [20]. Briefly, kidney cortex slices were homogenized for 2 min in 10 mM Hepes buffer 1 (300 mM D-mannitol, 5 mM EGTA, 12 mM MgCl_2_, pH 7.4). After staying for 20 min, the homogenate was centrifuged at 2,400×*g* for 15 min. The supernatant was centrifuged at 30,000×*g* for 30 min. The pellet was resuspended and homogenized in Hepes buffer 2 (150 mM D-mannitol, 2.5 mM EGTA, 12 mM MgCl_2_, pH 7.4) by a glass-Teflon homogenizer. The homogenate was again centrifuged at 2,400×*g* for 15 min and the supernatant was centrifuged at 30,000×*g* for 30 min. The final pellets were applied for Western blot analysis as described above and primary antibodies of rRST (1∶2000), rOCTN2 (1∶1000) (Sai-Chi Biotech. Beijing, P. R. China) and rat Na^+^-K^+^-ATPase (1∶1000) (Cell Signaling Technology, Boston, MA) were used, respectively.

### Statistical Analysis

Data were expressed as the mean ± standard error of the mean (SEM) deviation. Statistical analysis was performed by a one-way analysis of variance (ANOVA) followed by a Student-Newman-Kuel’s test. Differences were considered significant at *p*<0.05. The figures were obtained by the Statistical Analysis System (GraphPad Prism 4, GraphPad Software, Inc. San Diego, CA).

## Results

### Quercetin and Allopurinol Ameliorate Basic Characteristics of STZ-Treated Rats

As shown in Table 2, quercetin at 25, 50 and 100 mg/kg significantly increased body weight (27.5–46.3 g higher than STZ control group, *p*<0.001, 0.001 and 0.001), the highest dose decreased kidney-to-body weight ratio (61.8% of STZ control group, *p*<0.01) and blood glucose levels (74.6% of STZ control group, *p*<0.001) in STZ-induced diabetic rats compared with normal control group. STZ induced high levels of urine glucose (137-fold higher, *p*<0.001) and protein (2.44-fold higher, *p*<0.001) compared with normal control group (urine glucose: 3.39±0.48 mg/dL; urine protein: 17.30±1.41 mg/mL). 50 and 100 mg/kg quercetin also reduced urine levels of glucose (76.3 and 70.9% of STZ control group, *p*<0.05 and 0.05) and protein (71.7 and 63.0% of STZ control group, *p*<0.05 and 0.01), preventing glucosuria and proteinuria in STZ-treated rats. 10 mg/kg allopurinol exhibited its improvement on all these abnormalities in this model.

**Table 2:** Quercetin and allopurinol restore basic characteristics of streptozotocin (STZ)-treated rats.

Group	Dose (mg/kg)	Body weight(g)	Quot of kidney(×10^-2^)	Blood glucose(mg/dL)	Urine glucose(mg/dL)	Urine protein(×10^2^ mg/dL)
Normal Control		411.3 ± 6.5	0.63 ± 0.04	37.03 ± 2.15	3.39 ± 0.48	17.30 ± 1.41
STZ Control		177.1 ± 2.9^+++^	1.57 ± 0.16^+++^	297.94 ± 5.17^+++^	454.50 ± 31.45^+++^	42.21 ± 1.91^+++^
STZ + quercetin	100	223.4 ± 3.5^***^	0.97 ± 0.08^**^	222.32 ± 9.52^***^	322.49 ± 33.97^*^	26.58 ± 3.95^**^
STZ + quercetin	50	221.8 ± 2.3^***^	1.29 ± 0.16	278.54 ± 8.30	346.94 ± 11.74^*^	30.27 ± 3.25^*^
STZ + quercetin	25	204.6 ± 5.0^***^	1.50 ± 0.08	282.34 ± 3.55	397.15 ± 34.78	38.48 ± 0.86
STZ + allopurinol	10	221.3 ± 4.3^***^	1.05 ± 0.13^*^	218.33 ± 9.98^***^	316.75 ± 30.52^*^	24.67 ± 2.24^***^

The data are expressed as the means ± SEM (n = 8). ^+++^ *P*<0.001 *versus* normal control; ^*^ *P*<0.05, ^**^ *P*<0.01, ^***^ *P*<0.001 *versus* STZ control.

### Quercetin and Allopurinol Improve Renal Dysfunction and Hyperuricemia through Regulating Expression Levels of Renal Urate Transport-related Proteins in Stz-treated Rats

**Figure 1:**
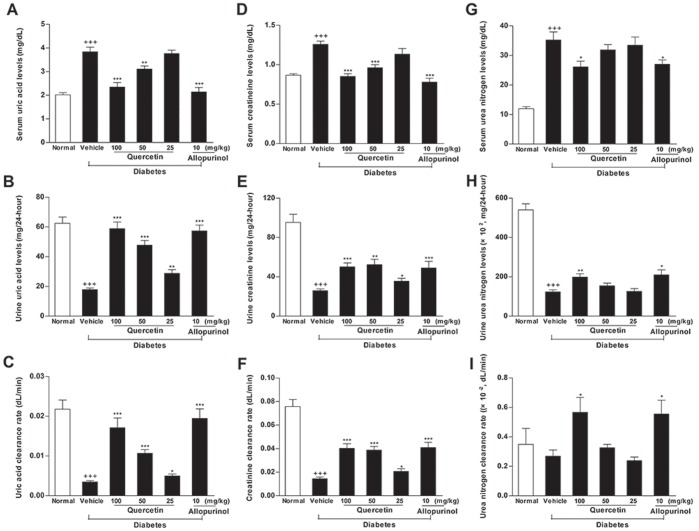
Quercetin and allopurinol restore streptozotocin (STZ)-induced hyperuricemia and kidney dysfunction in rats. Biochemical analyses showed uric acid (A-C), creatinine (D-F) and urea nitrogen (G-I) levels in serum and urine, as well as their clearance rates at 7 weeks after STZ injection in different groups of rats as indicated. The data are expressed as the means ± SEM (n = 8). ^+++^ *P*<0.001 *versus* normal control; *P<0.05, **P<0.01, ***P<0.001 *versus* STZ control.

As shown in Fig. 1A–I, STZ decreased total amount and clearance rate of uric acid (amount: 28.6% and clearance rate: 16.1% of normal control group, *p*<0.001 and 0.001), creatinine (amount: 27.2% and clearance rate: 19.2% of normal control group, *p*<0.001 and 0.001) and BUN (amount: 22.8% of normal control group, *p*<0.001) in 24-h urine, with the elevation of serum uric acid (1.9-fold higher than normal control group, *p*<0.001), creatinine (1.45-fold higher than normal control group, *p*<0.001) and BUN (2.95-fold higher than normal control group, *p*<0.001) concentrations in rats, indicating the shutdown of kidney function. STZ-treated rats receiving quercetin and allopurinol showed significant amelioration of renal dysfunction and hyperuricemia.

**Figure 2:**
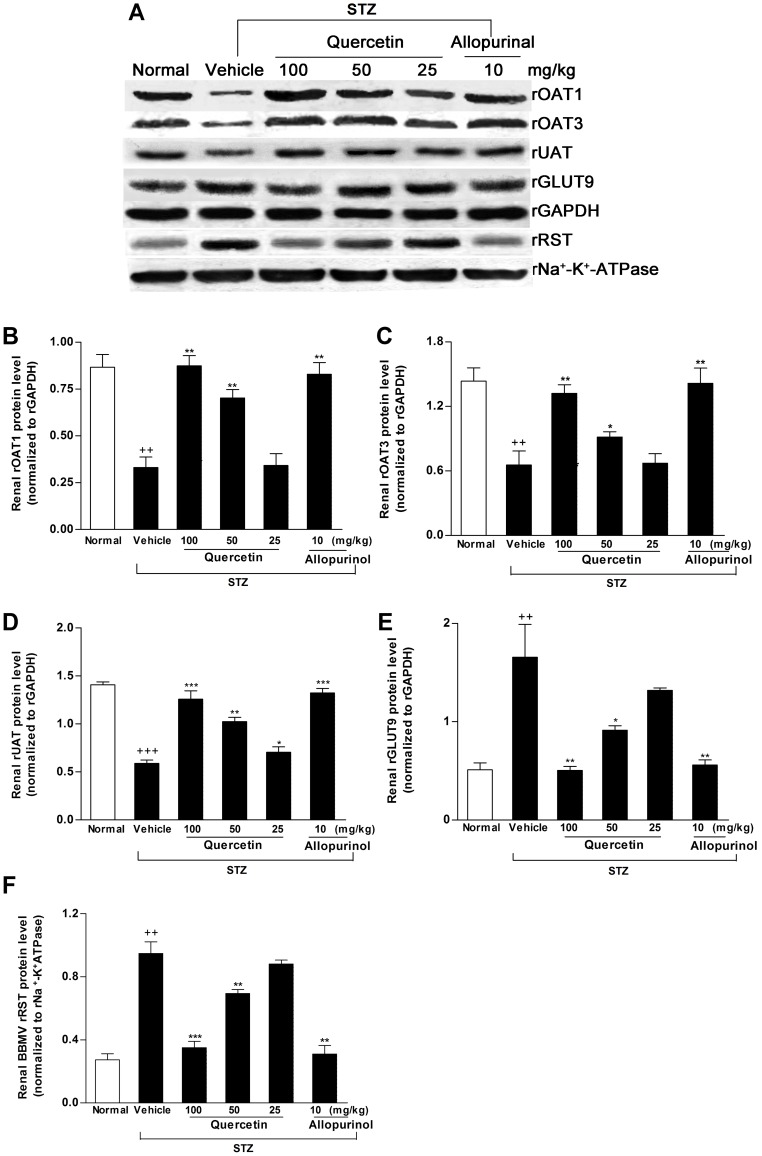
Quercetin and allopurinol regulate renal urate transport-related proteins in streptozotocin (STZ)-treated rats. Representative Western blot results (A) and graphic presentation showed renal protein expression of rOAT1 (B), rOAT3 (C), rUAT (D), rGLUT9 (E) and rRST (F) at 7 weeks after STZ injection in different groups of rats as indicated. The relative protein levels of rOAT1, rOAT3, rUAT and rGLUT9 were determined after normalization with rGAPDH. The relative BBMV rRST protein levels were normalized to rNa^+^-K^+^-ATPase. The data are expressed as the means ± SEM (n = 3–4). ^++^ *P*<0.01, ^+++^ *P*<0.001 *versus* normal control; *P<0.05, **P<0.01, ***P<0.001 *versus* STZ control.

**Figure 3:**
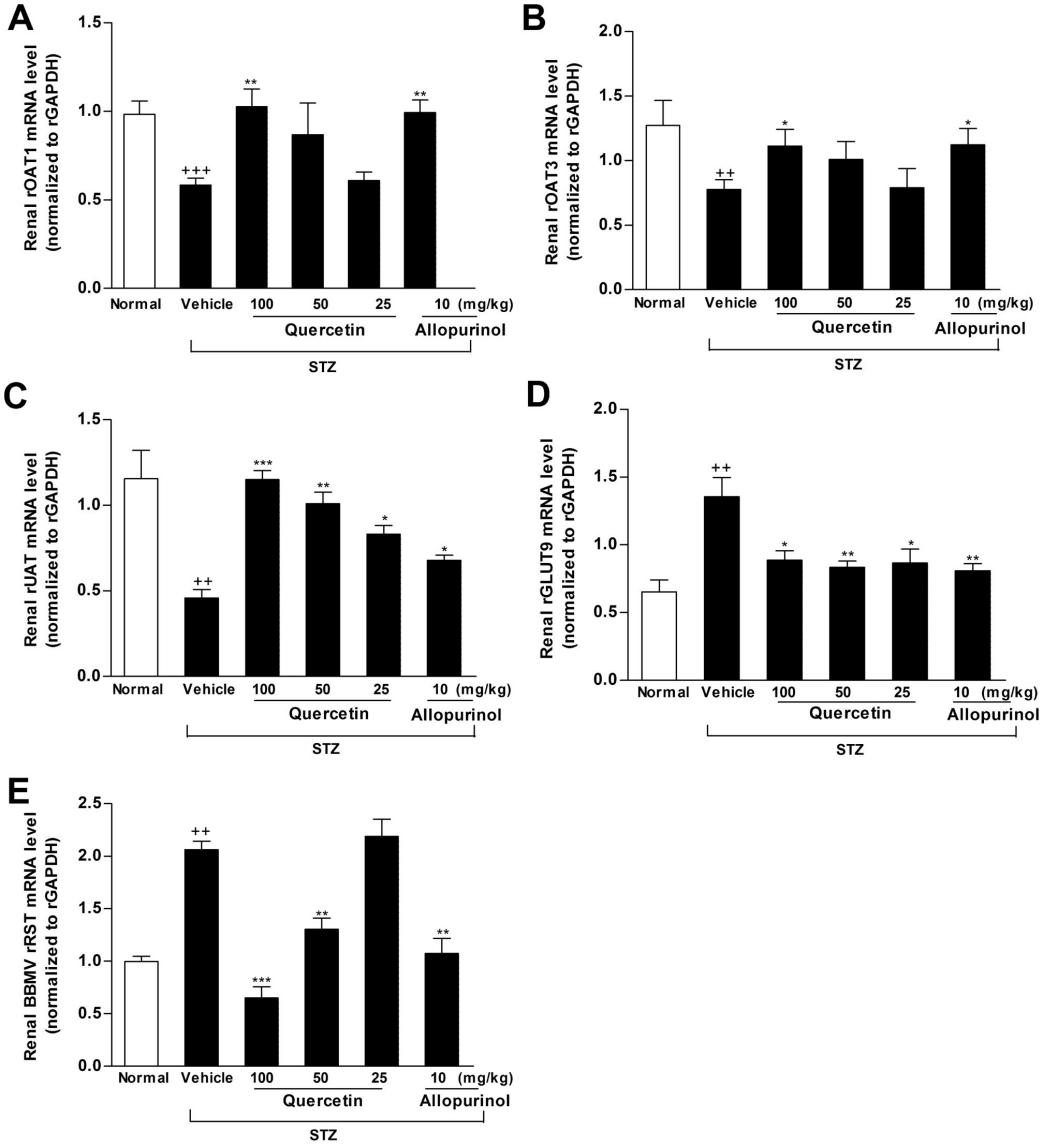
Quercetin and allopurinol regulate renal urate transport-related proteins mRNA in streptozotocin (STZ)-treated rats. Graphic presentation of renal mRNA levels by real-time PCR analysis of rOAT1 (A), rOAT3 (B), rUAT (C), rGLUT9 (D) and rRST (E) in different groups of rats as indicated. The relative mRNA levels were determined after normalization with rGAPDH. The data are expressed as the means±SEM (n = 3–4).^ ++^ *P*<0.01, ^+++^ *P*<0.001 *versus* normal control; *P<0.05, **P<0.01, ***P<0.001 *versus* STZ control.

Previous studies suggest the role of renal OATs, UAT, GLUT9 and RST in renal urate excretion and hyperuricemia [20], [34]. We examined the effects of quercetin and allopurinol on the expression levels of these renal transporters. Partly consistent with the previous reports in STZ-treated mice [35]–[36], the decreased mRNA and protein levels of renal rOAT1 (mRNA: *p*<0.001; protein: *p*<0.01), rOAT3 (mRNA and protein: *p*<0.01) and rUAT (mRNA: *p*<0.01; protein: *p*<0.001) with the increased mRNA and protein levels (*p*<0.01) of rGLUT9 and rRST were observed in STZ-induced diabetic rats compared with normal control group (Fig. 2A–F, 3A–F). Quercetin and allopurinol restored STZ-induced expression abnormality of these renal urate transporters at mRNA and protein levels in rats (Fig. 2A–F, 3A–E), and 100 mg/kg quercetin reached the significant improvement similar to allopurinol, further demonstrating their anti-hyperuricemic effects.

### Quercetin and Allopurinol Reduce Dyslipidemia and Renal Lipid Accumulation through Regulating Lipid Metabolism-related Genes in Stz-treated Rats

**Figure 4:**
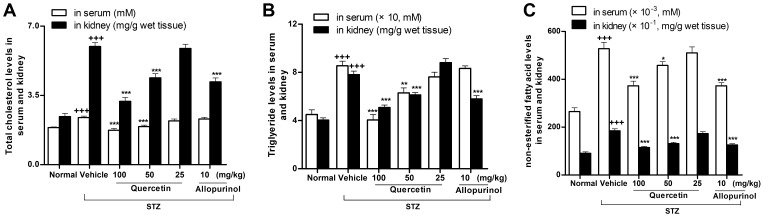
Quercetin and allopurinol reduce lipid levels in streptozotocin (STZ)-treated rats. Biochemical analyses showed serum and kidney cortex levels of TC (A), TG (B) and NEFA (C) at 7 weeks after STZ injection in different groups of rats as indicated. The data are expressed as the means ± SEM (n = 8). ^+++^ *P*<0.001 *versus* normal control; *P<0.05, **P<0.01, ***P<0.001 *versus* STZ control.

**Figure 5:**
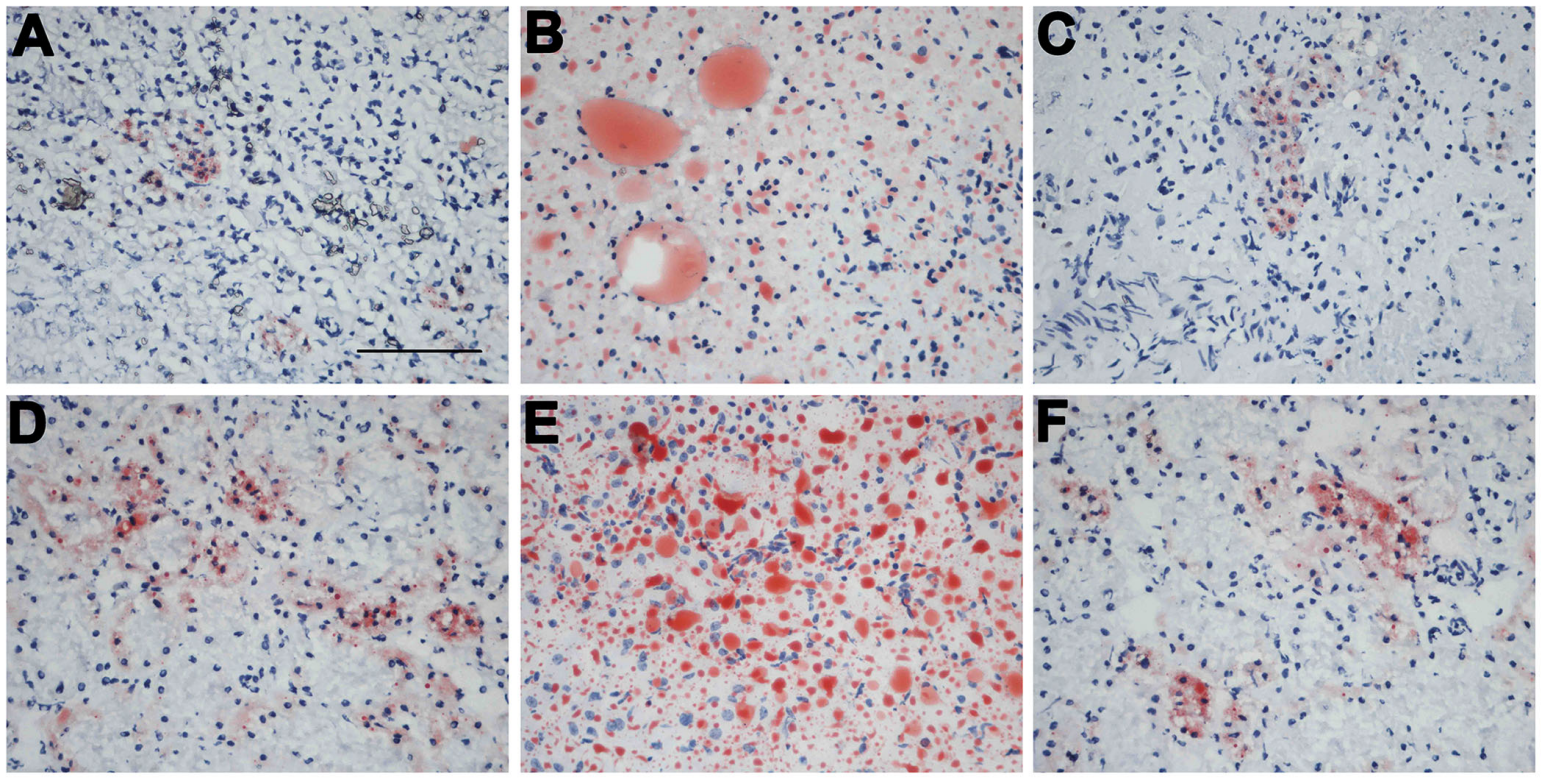
Quercetin and allopurinol reduce renal lipid accumulation in streptozotocin (STZ)-treated rats. Representative micrographs demonstrated kidney histology in different groups of rats. Kidney sections were stained with oil-red O. Normal control (A), STZ alone (B), STZ plus 100 mg/kg quercetin (C), STZ plus 50 mg/kg quercetin (D), STZ plus 25 mg/kg quercetin (E) and STZ plus 10 mg/kg allopurinol (F). Bar = 50 µm in Fig. 5A.

We next examined the effects of quercetin and allopurinol on dyslipidemia and renal lipid accumulation in STZ-treated rats. The 12-h fasting alters lipid metabolism-related genes and induces lipid accumulation in the kidney of mice [37]. In this study, we detected serious lipid accumulation in the kidney of STZ-treated rats compared with normal control group (Fig. 4–5). As shown in Fig. 4A–C, STZ induced elevation of serum and kidney levels of TC (serum: 1.3-fold; kidney: 2.5-fold higher than normal control group), TG (serum and kidney: 1.9-fold higher than normal control group) and NEFA (serum and kidney: 2.0-fold higher than normal control group) in rats, which were restored effectively by the treatment of 50 and 100 mg/kg quercetin (TC in serum and kidney: *p*<0.001; TG in serum: *p*<0.01 and 0.001, in kidney: *p*<0.001; NEFA in serum: *p*<0.01 and 0.001, in kidney: *p*<0.001 and 0.001). 10 mg/kg allopurinol restrained three types of lipids in the kidney of STZ-treated rats. These results were consistent with and supported by renal lipid deposition improvement in renal tubular epithelial cells of STZ-treated rats receiving quercetin or allopurinol assessed by oil red O-stain analysis (Fig. 5A–F).

**Figure 6:**
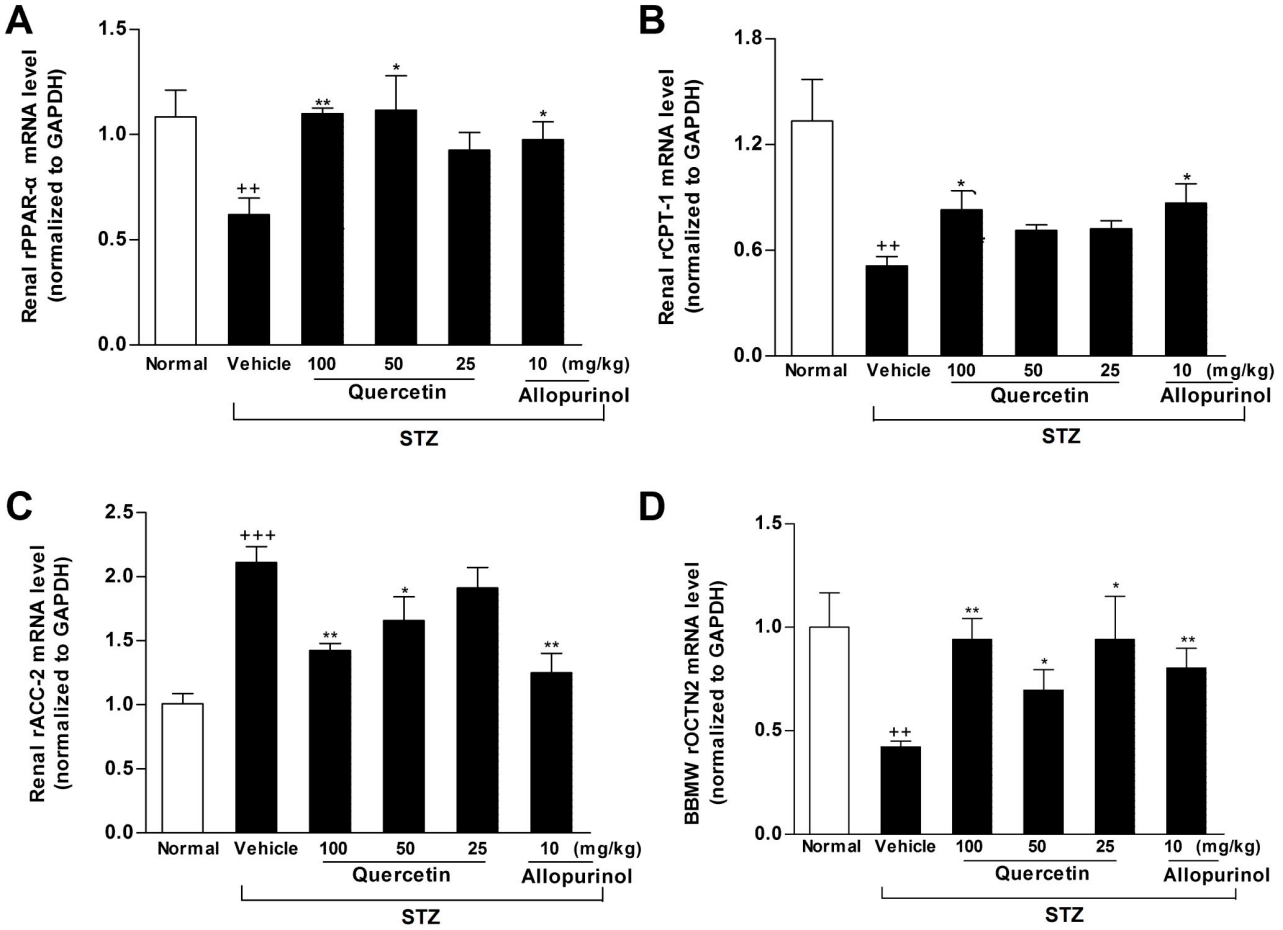
Quercetin and allopurinol regulate renal mRNA levels of lipid metabolism-related genes in streptozotocin (STZ)-treated rats. Graphic presentation of renal mRNA levels by real-time PCR analysis of rPPAR-α (A), rCPT1 (B), rACC2 (C) and rOCTN2 (D) at 7 weeks after STZ injection in different groups of rats as indicated. The relative mRNA levels were determined after normalization with rGAPDH. The data are expressed as the means ± SEM (n = 3–4). ^+++^ *P*<0.001 *versus* normal control; *P<0.05, **P<0.01, ***P<0.001 *versus* STZ control.

**Figure 7:**
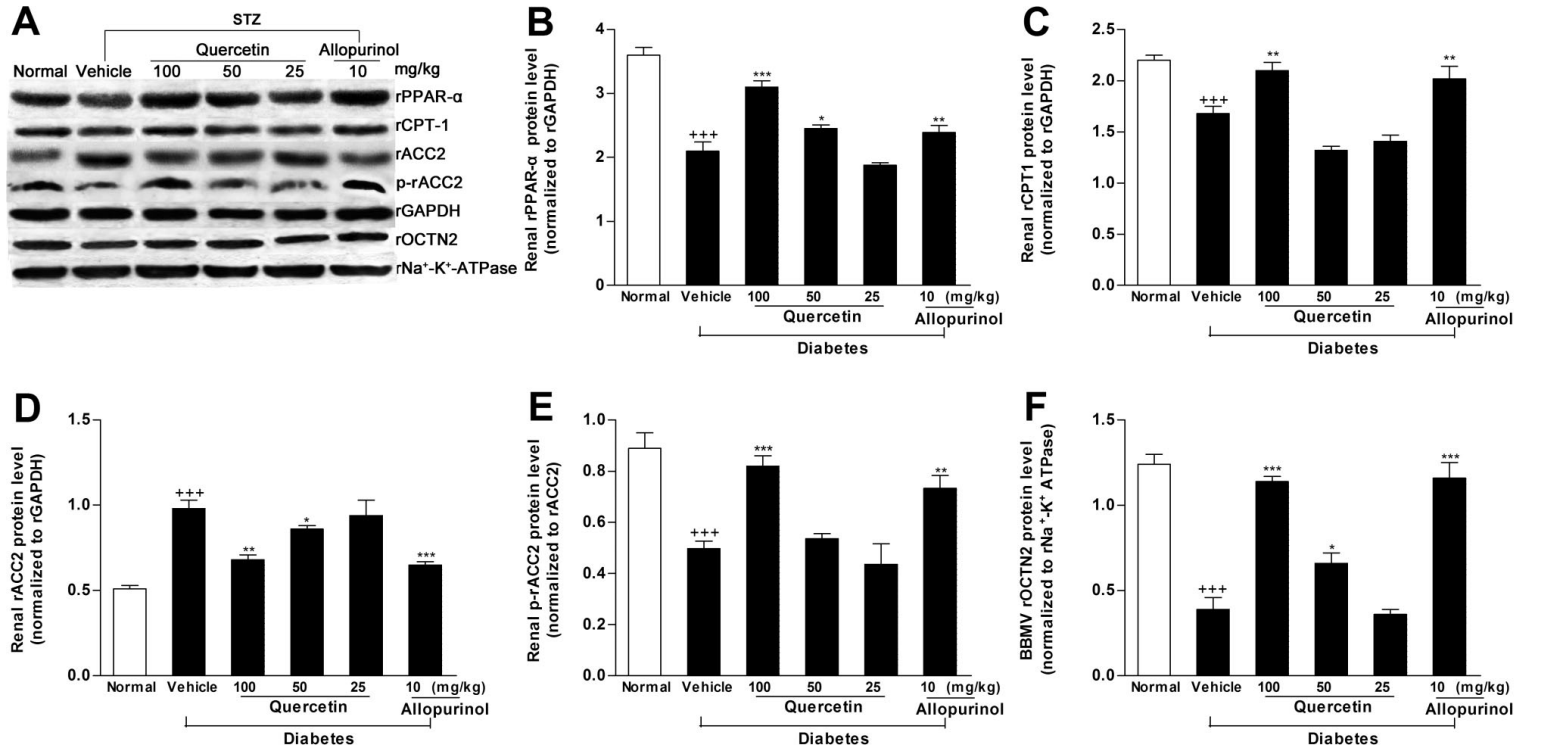
Quercetin and allopurinol regulate renal expression of lipid metabolism-related genes protein levels in streptozotocin (STZ)-treated rats. Representative Western blot results (A) and graphic presentation showed renal protein expression of rPPAR-α (B), rCPT1 (C), rACC2 (D), p-rACC2 (E) and rOCTN2 (F) in different groups of rats as indicated. Relative protein levels of rPPAR-α, rCPT1 and rACC2 were determined after normalization with rGAPDH. The relative renal BBMV rOCTN2 protein levels were normalized to rNa^+^-K^+^-ATPase. The data are expressed as the means ± SEM (n = 3–4). ^++^ *P*<0.01, ^+++^ *P*<0.001 *versus* normal control; *P<0.05, **P<0.01, ***P<0.001 *versus* STZ control.

**Figure 8:**
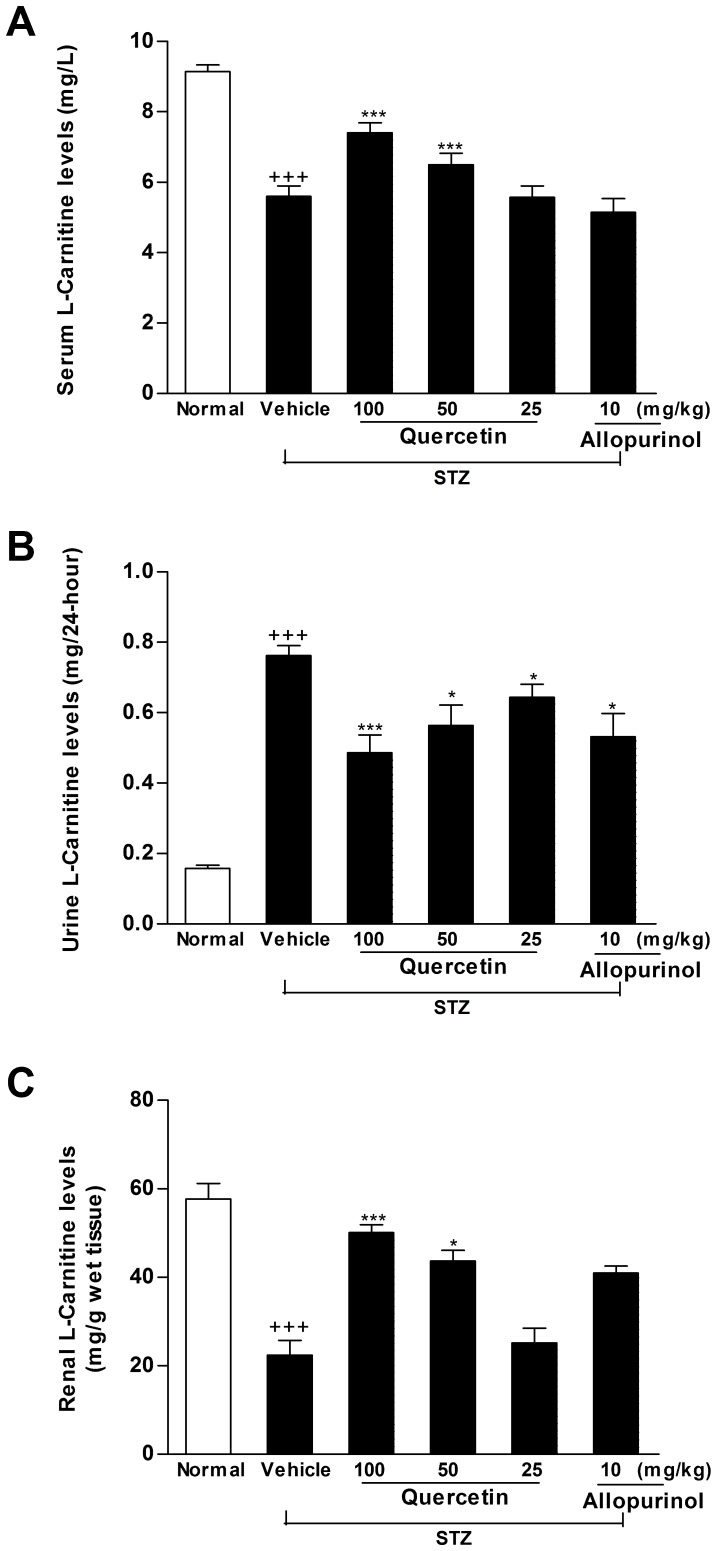
Quercetin and allopurinol regulate renal L-carnitine levels in streptozotocin (STZ)-treated rats. Biochemical analyses showed L-carnitine levels in serum (A) and urine (B) and kidney (C) at 7 weeks after STZ injection in different groups of rats as indicated. The data are expressed as the means ± SEM (n = 8). ^+++^ *P*<0.001 *versus* normal control; *P<0.05, **P<0.01, ***P<0.001 *versus* STZ control.

PPAR-α is a key regulator of its target genes CPT1 and OCTN2, both of which are involved in mitochondrion fatty acid β-oxidation [35]. OCTN2 is capable of transporting L-carnitine [36], which acts as an obligatory co-factor for fatty acid β-oxidation by facilitating transportation of long-chain fatty acids across mitochondrial membrane. CPT1 is demonstrated to mediate transportation of long-chain fatty acids across mitochondria outer membrane by binding them to L-carnitine [38]. Therefore, we investigated the expression levels of these renal lipid metabolism-related genes in STZ-treated rats. Renal rPPAR-α, rCPT1 and rOCTN2 mRNA (rPPAR-α: *p*<0.01; rCPT1: *p*<0.01; rOCTN2: *p*<0.01) (Fig. 6A–B, D) and protein (rPPAR-α: *p*<0.001; rCPT1: *p*<0.001; rOCTN2: *p*<0.001) (Fig. 7A–C, F) levels were down-regulated in STZ-induced diabetic rats compared with normal control group. Of note, the decreased L-carnitine levels in serum (61% of normal control group, *p*<0.001) and kidney (38.8% of normal control group, *p*<0.001) as well as the increased L-carnitine levels in urine (4.8-fold higher than normal control group, *p*<0.001) were detected in this model (Fig. 8A–C). ACC catalyzes biotin-dependent carboxylation of acetyl-CoA to produce malonyl-CoA. ACC2-generated malonyl-CoA functions as inhibitor of CPT1 activity and the transfer of fatty acyl group through the carnitine/palmitoyl shuttle system to inter mitochondria for β-oxidation [39]. Accordingly, rACC2 mRNA (2.1-fold higher than normal control group, *p*<0.001) (Fig. 6C) and protein (1.9-fold higher than normal control group, *p*<0.001) (Fig. 7A, D) levels were substantially increased, whereas, p-rACC2 (Ser 219/Ser 221) protein levels (22.3% of normal control group, *p*<0.001) (Fig. 7A, E) was suppressed in the kidney of STZ-treated rats. These results demonstrated disorders of these lipid regulators in this model. Of interest, administration of 50 and 100 mg/kg quercetin and 10 mg/kg allopurinol effectively elevated rPPAR-α (mRNA: 50 mg/kg quercetin *p*<0.05, 100 mg/kg quercetin *p*<0.01, 10 mg/kg allopurinol *p*<0.05; protein: 50 mg/kg quercetin *p*<0.05, 100 mg/kg quercetin *p*<0.001, 10 mg/kg allopurinol *p*<0.01) (Fig. 6A, 7A–B), correspondingly, restrained down-expression of rCPT1 (mRNA: 100 mg/kg quercetin *p*<0.05, 10 mg/kg allopurinol *p*<0.05; protein: 100 mg/kg quercetin *p*<0.01, 10 mg/kg allopurinol *p*<0.01) (Fig. 6B, 7A,C) and rOCTN2 (mRNA: 50 mg/kg quercetin *p*<0.05, 100 mg/kg quercetin *p*<0.01, 10 mg/kg allopurinol *p*<0.01; protein: 50 mg/kg quercetin *p*<0.05, 100 mg/kg quercetin *p*<0.001, 10 mg/kg allopurinol *p*<0.001) (Fig. 6D, 7A, F) in the kidney of STZ-treated rats compared with STZ control group. They also increased kidney and serum L-carnitine levels and reduced urine L-carnitine levels in this model (Fig. 8). In addition, quercetin and allopurinol restored STZ-induced alteration in renal rACC2 expression (mRNA: 50 mg/kg quercetin *p*<0.05, 100 mg/kg quercetin *p*<0.01, 10 mg/kg allopurinol *p*<0.01; protein: 50 mg/kg quercetin *p*<0.05, 100 mg/kg quercetin *p*<0.01, 10 mg/kg allopurinol *p*<0.001) and p-rACC2 (50 mg/kg quercetin *p*<0.05, 100 mg/kg quercetin *p*<0.001, 10 mg/kg allopurinol *p*<0.01) levels in rats (Fig. 6C, 7A, D, E). These results suggested that quercetin and allopurinol improved lipid accumulation through the regulation of lipid metabolism in STZ-treated rats.

### Quercetin and Allopurinol Ameliorate Kidney Inflammation by Blocking the NLRP3 Inflammasome Activation in STZ-treated Rats

**Figure 9:**
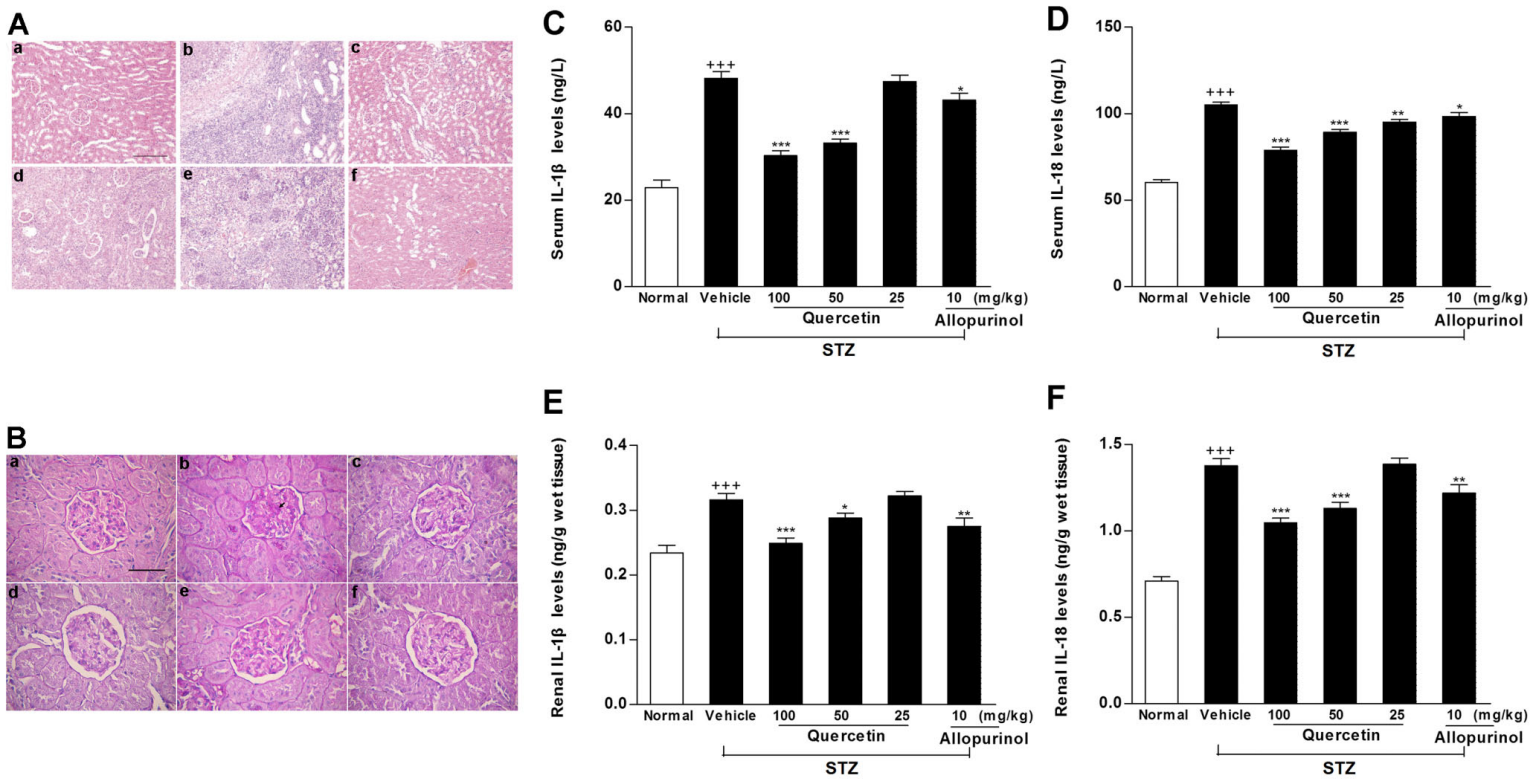
Quercetin and allopurinol attenuate inflammation in streptozotocin (STZ)-treated rats. HE stain (A) analyses showed inflammatory cell infiltration and PAS-D stain (B) analyses showed kidney structures in different groups of rats. Normal control (a), STZ alone (b), STZ plus 100 mg/kg quercetin (c), STZ plus 50 mg/kg quercetin (d), STZ plus 25 mg/kg quercetin (e) and STZ plus 10 mg/kg allopurinol (f); Bar = 50 µm in Fig. 9Aa and Bar = 15 µm in Fig. 9Ba. Biochemical analyses showed serum and kidney levels of IL-1β (C, E) and IL-18 (D, F) at 7 weeks after STZ injection in different groups of rats as indicated. The data are expressed as the means ± SEM (n = 8). ^+++^ *P*<0.001 *versus* normal control; *P<0.05, **P<0.01, ***P<0.001 *versus* STZ control.

High urate level and lipid disorder are associated with inflammation [4], [7], [11], [12]. In parallel with hyperuricemia and lipid accumulation, STZ-treated rats showed inflammatory cell infiltration in glomerulus and renal tubular (Fig. 9A) and the destroyed kidney structure as mesangial expansion (Fig. 9B). These kidney pathological changes in this model were ameliorated by the treatment of quercetin and allopurinol (Fig. 9A–B).

**Figure 10:**
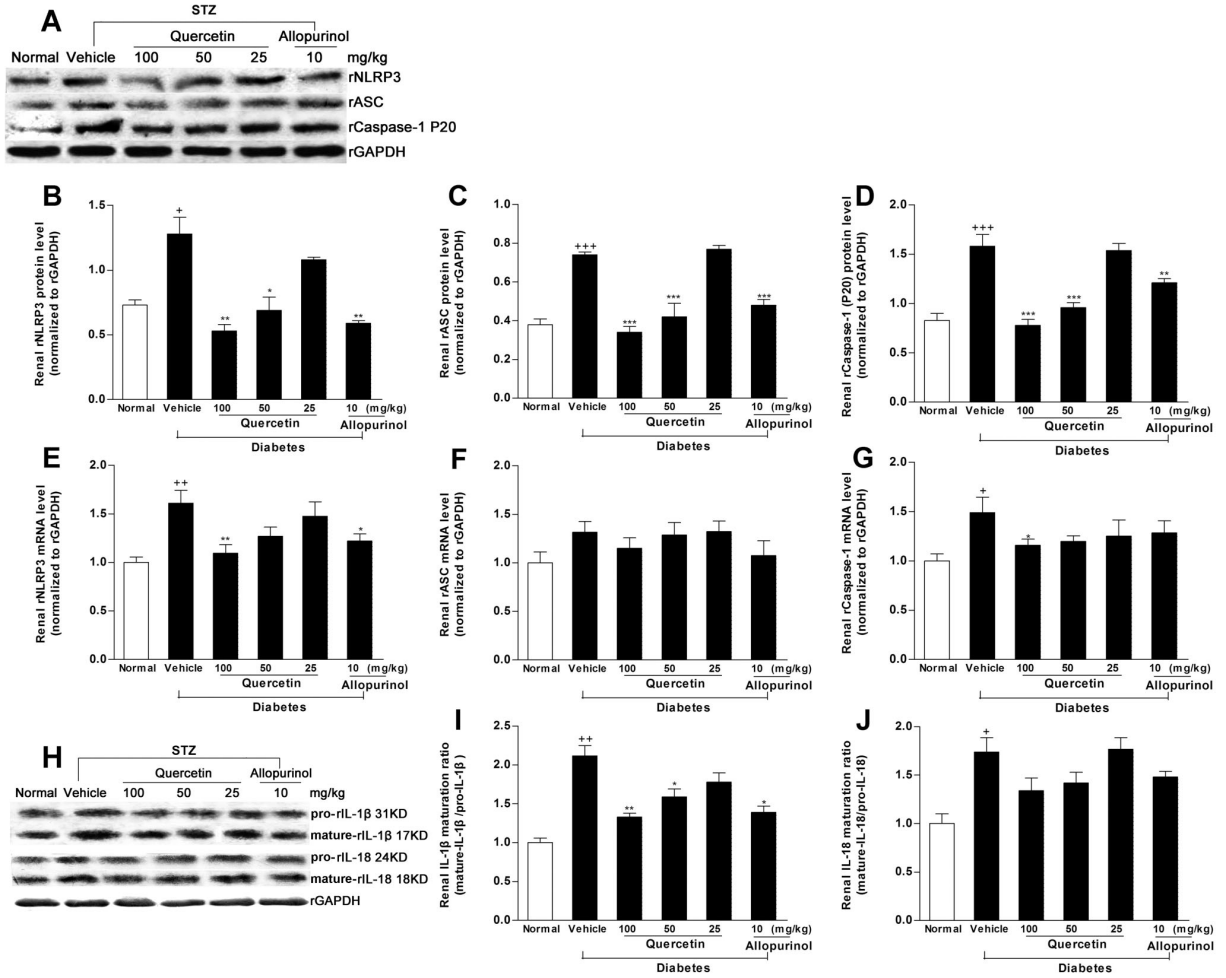
Quercetin and allopurinol inhibit renal NALP3 inflammasome activation in streptozotocin (STZ)-treated rats. Representative Western blot results (A) and graphic presentation showed renal protein expression of rNALP3 (B), rASC (C) and rCaspase-1 (D) in different groups of rats as indicated. Relative protein levels of rNALP3, rASC and rCaspase-1 were determined after normalization with rGAPDH. For rCaspase-1, the active subunit of P20 was detected. The data are expressed as the means ± SEM (n = 3–4). ^+^ *P*<0.05, ^+++^ *P*<0.001 *versus* normal control; *P<0.05, **P<0.01, ***P<0.001 *versus* STZ control. Graphic presentation of renal mRNA levels by real-time PCR analysis of rNALP3 (E), rASC (F) and rCaspase-1 (G) at 7 weeks after STZ injection in different groups of rats as indicated. The relative mRNA levels were determined after normalization with rGAPDH. The data are expressed as the means ± SEM (n = 3–4). ^+++^ *P*<0.001 *versus* normal control; *P<0.05, **P<0.01, ***P<0.001 *versus* STZ control. Representative Western blot results (H) and graphic presentation showed renal maturation ratio of IL-1β (I) and IL-18 (J) in different groups of rats as indicated. The data are expressed as the means ± SEM (n = 3–4). ^+^ *P*<0.05, ^++^ *P*<0.01 *versus* normal control; *P<0.05, **P<0.01 *versus* STZ control.

To provide mechanistic insights into nephroprotective efficacy of quercetin and allopurinol in STZ-treated rats, we investigated their effects on renal NLRP3 inflammasome activation, because recent studies suggest that this inflammasome is involved in kidney injury [13], [14]. Western blot analyses demonstrated that the expression levels of renal rNLRP3 (*p*<0.05), rASC (*p*<0.001) and rCaspase-1 (for Western blot analysis of rCaspase-1, we detected the active subunit P20) (*p*<0.001) were increased in STZ-treated rats compared with normal control group (Fig. 10A–D). PCR analyses found the induced elevation of renal mRNA levels of rNLRP3 (*p*<0.01) and rCaspase-1(*p*<0.05) in this model compared with normal control group (Fig. 10E–G). The activated caspase-1 contributes to the maturation of IL-1β and IL-18 [13], [14]. Consistent with the increased ratio (2.1-fold higher than normal control rats) of mature-IL-1β (17 kD)/pro-IL-1β (31 kD), the similar maturative effect (1.5-fold higher than normal control group) induced by Caspase-1 was also observed in the kidney of STZ-treated rats by analyses of mature-IL-18 (18 kD)/pro-IL-18 (24 kD) (Fig. 10H–J). Accordingly, serum and kidney concentrations of IL-1β (serum: 2.1-fold; kidney: 1.4-fold higher than normal control rats) and IL-18 (serum: 1.7-fold; kidney: 1.9-fold higher than normal control rats) were remarkably elevated in STZ-treated rats (Fig. 9C–F). These results might provide evidence for a role of the NLRP3 inflammasome activation in STZ-induced renal inflammation under pathologic condition of hyperuricemia and dyslipidemia in rats. We found that this over-expression of renal rNLRP3, rASC and rCaspase-1 (P20) in STZ-treated rats was restrained dramatically by the treatment of 100 mg/kg quercetin (rNLRP3: mRNA *p*<0.01, protein *p*<0.01; rASC: protein *p*<0.001; rCaspase-1: mRNA *p*<0.05, protein *p*<0.001), and 10 mg/kg allopurinol (rNLRP3: mRNA *p*<0.05, protein *p*<0.01; rASC: protein *p*<0.001; rCaspase-1: protein *p*<0.01) compared with STZ control group (Fig. 10A–G). 100 mg/kg quercetin and 10 mg/kg allopurinol also reduced serum and kidney levels of IL-1β (serum: 100 mg/kg quercetin *p*<0.001, 10 mg/kg allopurinol *p*<0.05; kidney: 100 mg/kg quercetin *p*<0.001, 10 mg/kg allopurinol *p*<0.01) and IL-18 (serum: 100 mg/kg quercetin *p*<0.001, 10 mg/kg allopurinol *p*<0.05; kidney: 100 mg/kg quercetin *p*<0.001, 10 mg/kg allopurinol *p*<0.01) (Fig. 9C–F) in this model, with the restoration of renal mature-IL-1β/pro-IL-1β changes (100 mg/kg quercetin *p*<0.01, 50 mg/kg quercetin *p*<0.05, 10 mg/kg allopurinol *p*<0.05) but not renal mature-IL-18/pro-IL-18 (Fig. 10H–J). By reducing urate and lipid levels, the NLRP3 inflammasome activation may be suppressed by the treatment with quercetin and allopurinol to protect STZ-induced kidney injury.

## Discussion

Uric acid is a mediator of diabetic kidney injury [5], [6], [40]. The present study also observed hyperuricemia and kidney function decline in STZ-induced diabetic rats. Partly consistent with the previous reports in STZ-treated mice [41], [42], deregulation of renal rOAT1, rOAT3, rUAT, rGLUT9 and rRST may increase urate reabsorption and decrease urate secretion, subsequently resulting in renal urate under-excretion and serum urate elevation in STZ-induced diabetic rats. Chronic treatment of quercetin and allopurinol restored expression abnormality of these renal urate transport-related proteins to enhance urate excretion and reduce serum urate levels in STZ-treated rats, which were parallel with our previous study in fructose-induced hyperuricemia and kidney injury in rats [20]. These observations demonstrate that urate-lowering efficacy of quercetin and allopurinol may improve renal function and injury in STZ-treated rats.

Hyperuricemia is associated with dyslipidemia [16]. The altered lipid metabolism is suggested to be responsible for the progression of diabetic renal disease [3], [43]. PPAR-α mediates lipid metabolism and inflammation [30], [44], [45] and plays a role in the development of diabetic kidney injury [46], [47]. Down-regulation of renal PPAR-α was detected in STZ-induced diabetic rats in the present study. Additionally, PPAR-α regulates CPT1 and OCTN2 involved in mitochondrion fatty acid β-oxidation [35]. Experimental studies have demonstrated that L-carnitine reduces serum TG and TC levels and improves lipid metabolism in STZ-induced diabetic rats [48], relieves lipid overload in obese rodents with diabetes [49] and prevents renal injury [50]. The present study found that STZ-induced diabetic kidney injury was accompanied with down-regulation of renal rOCTN2, an important transporter for L-carnitine reabsorption, and reduction of renal and serum L-carnitine levels, showing carnitine deficit in rats compared with normal control group. High glucose increases levels of malonyl-CoA, a CPT1 inhibitor and diminishes fatty acid β-oxidation through ACC activation [51]. Consistent with the previous studies in STZ-induced diabetic rats [52] and patients with type 2 diabetes [3], the present study demonstrated the decreased expression levels of rCPT1 and p-rACC2 (Ser 219/Ser 221), and the increased expression levels of rACC2 in the kidney of STZ-induced diabetic rats compared with normal control group. These results indicate that dysexpression of these renal lipid metabolism-related genes, as well as carnitine deficiency may be involved in fatty acid β-oxidation reduction, triggering hypertriglyceridemia to cause renal lipid accumulation injury in STZ-treated rats. It is known that 12 h of fasting alters apolipoprotein B-containing lipoproteins levels and induces TG but not cholesterol accumulation in mice [37].Therefore, STZ-induced kidney lipid metabolism disorder and lipid accumulation in rats superimposed on renal injury incurred with 12 h fasting in this study. The activation of PPAR-α attenuates or inhibits lipotoxicity and inflammation in diabetic microvascular disease [47]. Quercetin and allopurinol improve dyslipidemia in fructose-fed rats [20], [21], the former reduces hepatic fat accumulation in Western-style diet-fed mice [53]. Interestingly, quercetin and allopurinol ameliorated abnormality of renal rPPAR-α, rCPT1, rOCTN2, rACC2 and p-rACC2 expressions and L-carnitine levels, subsequently resulting in the attenuation of renal lipid profile change and lipid accumulation in this model. These results may suggest a possible role of urate-lowering agents quercetin and allopurinol to suppress the superimposed renal lipid metabolism disorder that occurs in STZ-treated rats in this study.

The NLRP3 inflammasome is a multiprotein complex to control caspase-1 activation step that is a prerequisite for the maturation of IL-1β and IL-18. Recent studies document the role of the NLRP3 inflammasome-caspase-1-IL-1β/IL-18 axis in kidney disease [13], [14], [15]. In the diabetic state, elevation of circulating and locally produced proinflammatory cytokines leads to the persistence of inflammation, which correlates the development and progression of diabetic kidney injury [2], [8], [9]. Thus, inflammation is considered as a cardinal pathogenetic mechanism in diabetic kidney injury [54]. Uric acid and lipid danger signals are proposed to be important in activating the NALP3 inflammasome [7], [11], [12]. Consistent with renal inflammatory symptom observed in STZ-induced diabetic rats with hyperuricemia and renal lipid accumulation, the present study for the first time demonstrated renal NLRP3 inflammasome activation evidenced by over-expression levels of renal rNLRP3, rASC and rCaspase-1, especially significantly increased levels of active form of Caspase-1(P20), resulting in the maturation of IL-1β and IL-18 in STZ-induced diabetic rats compared with normal control group. The NLRP3 inflammasome activation and subsequent maturation and secretion of IL-1β and IL-18 initiated further pro-inflammatory events, producing kidney damage of STZ-induced diabetic rats. Inflammation is demonstrated to be associated with lipid metabolism in diabetic subjects [55]. IL-1β and IL-18 are reported to affect metabolism of lipid and glucose [56], [57]. Therefore, STZ-induced NLRP3 inflammasome activation may further deteriorate kidney lipid metabolism in rats. This finding of renal NLRP3 inflammasome activation may provide a mechanism underlying renal inflammation and lipid accumulation after STZ induction diabetes and may be a significant contributory factor for diabetic kidney injury. Of note, additional studies have indicated that not only NLRP3, but also NLRP1 [58], NLRC4 [NLR (Nod-like receptor) family CARD-containing 4] [59] and AIM2 (absent in melanoma 2) [60], [61] form the inflammasomes that drive inflammation in response to a wide variety of molecular patterns. Similar to NLRP3, NLRP1 and AIM2 indirectly recruit and thereby activate Caspase-1 through ASC. NLRC4 regulates Caspase-1 activation and IL-1β processing in response to infection with various gram-negative bacteria. Stimulation with STZ may induce renal NLRP1, NLRC4 and AIM2 inflammasomes assembly and Caspase-1 activation in rats. Dysregulation of these inflammasomes may contribute to renal inflammation and lipid metabolism in STZ-treated rats. Further studies for the detection of the activation of renal NLRP1, NLRC4 and AIM2 inflammasomes in diabetic state are warranted.

Quercetin is capable of preventing high glucose-induced IL-1β expression [62] and STZ-induced diabetic kidney injury in rats [27]. Allopurinol successfully treats perforating collagenosis of diabetes and renal failure [23]. Recently, allopurinol is found to inhibit the NLRP3 inflammasome activation [7], [19] and control IL-1β production in inflammasome-deficient mice [7]. In this study, the increased ratio of kidney weight to body weight and renal inflammation in STZ-treated rats were significantly restored by the treatment of quercetin and allopurinol. Quercetin and allopurinol were firstly confirmed to suppress renal NLRP3 inflammasome activation characterized by down-regulation of the NLRP3 and therefore reduction of Caspase 1 in STZ-treated rats. Subsequently, Caspase-1 inhibition by quercetin and allopurinol prevented kidney IL-1β and IL-18 release and over-production triggered by hyperuricemia and dyslipidemia in this model. These results of quercetin and allopurinol-produced suppression of renal NLRP3 inflammasome activation were parallel with their reductions of renal lipid accumulation in STZ-treated rats. These findings suggest that urate-lowering efficacy of quercetin and allopurinol may contribute to the suppression of the NLRP3 inflammasome activation involved in the normalization of IL-1β and IL-18 levels and reduction of renal lipid accumulation, which may benefit to ameliorate kidney injury of STZ-treated rats.

It is known that STZ is acutely nephrotoxic, leading to DNA damage and cellular proliferation in rats up to 3 weeks after 60 mg/kg STZ iv administration [63], indicating that studies examining the effects of drug treatments on the development of diabetic nephropathy should not be started until at least 3 weeks after STZ when the kidney has recovered from the acute mild nephrotoxic effects of STZ [63]–[64]. On the other hand, the NLRP3 inflammasome activation is involved in renal acute tubular necrosis [14], [40]. Moreover, *in vivo* ischemia/reperfusion acutely increases renal cortical cholesterol ester, but not free cholesterol levels in the kidney of mice [65]. Renal injury also induces dramatic TG accumulation in proximal tubules/renal cortex of CD-1 mice [66]. These observations indicate that renal toxic injury and acute tubular necrosis are more likely related to acute kidney lipid overload [65]–[66]. Moreover, 12 h of fasting induces lipid accumulation and alters genes involved in lipid metabolism in the kidney of mice [37]. In this study, it is incompletely understood how renal acute injury, lipid accumulation and inflammation interact and whether the combined effects on STZ-induced nephrotoxicity in rats are additive or synergistic. But, renal acute injury, lipid accumulation and inflammation appear as a dangerous mix, and there may be important superimposed effects of either condition with regard to crucial triggers of nephrotoxicity in this model. In the present study, the treatment of quercetin and allopurinol was started on the 4th day after STZ injection in rats. So it is quite possible that the restoration of quercetin and allopurinol on the superimposed nephrotoxicity in STZ-diabetic rats should be attributed to additive or synergistic effects rather than the treatment per se.

In conclusion, the present study demonstrated that STZ induced renal expression abnormality of urate transport-related proteins and lipid metabolism-related genes, showing hyperuricemia and dyslipidemia in diabetic nephropathy rats. Furthermore, renal NLRP3 inflammasome activation was observed in STZ-treated rats, resulting in elevation of IL-1β and IL-18, with subsequent deterioration of renal injury. These findings may provide evidence for the possible association between renal NLRP3 inflammasome activation and lipid metabolism disorder to superimpose causes of STZ-induced nephrotoxicity with hyperuricemia in rats. The treatment of quercetin and allopurinol with anti-hyperuricemic and anti-dyslipidemic effects may prevent the superimposed nephrotoxicity induced by STZ in rats. Although the detailed mechanisms of the nephroprotective effects of quercetin and allopurinol on diabetic nephrotoxicity remain to be fully defined, the present study should draw further attention to the utility of urate-lowering agents such as quercetin and allopurinol to gain new insights into the prevention of kidney injury.
